# Supplementary material for: Macroscopic phase separation of superconductivity and ferromagnetism in Sr0.5Ce0.5FBiS2−xSex revealed by μSR
Source: Sci Rep. 2017 Dec 12;7:17370. doi: 10.1038/s41598-017-17637-y (PMC5727222; doi:10.1038/s41598-017-17637-y)
Supplement: Supplementary file 1 — Supplementary Information [file 41598_2017_17637_MOESM1_ESM.pdf]

SUPPLEMENTARY INFORMATION

Macroscopic phase separation of superconductivity and ferromagnetism in $Sr_{0.5}Ce_{0.5}FBiS_{2-x}Se_x$ revealed by μSR

A.M. Nikitin[1], V. Grinenko[2,3], R. Sarkar[2], J.-C. Orain[4], M. Salis[1], J. Henke[1], Y.K. Huang[1], H.-H. Klauss[2], A. Amato[4] and A. de Visser[1]

[1]*Van der Waals - Zeeman Institute, University of Amsterdam, 1098 XH Amsterdam, The Netherlands*
[2]*Institute of Solid State and Materials Physics, Technical University Dresden, 01062 Dresden, Germany*
[3]*Leibniz Institute for Solid State and Materials Research (IFW), 01069 Dresden, Germany*
[4]*Laboratory for Muon-Spin Spectroscopy, Paul Scherrer Institute, 5232 Villigen PSI, Switzerland*

Content

Part 1: Characterization of the $Sr_{0.5}Ce_{0.5}FBiSSe$ compound

1. X-ray diffraction
2. Magnetic susceptibility and magnetization of $Sr_{0.5}Ce_{0.5}FBiSSe$
3. Specific heat of $Sr_{0.5}Ce_{0.5}FBiSSe$
4. Ac susceptibility of $Sr_{0.5}Ce_{0.5}FBiSSe$
5. Electrical resistivity and upper critical field of $Sr_{0.5}Ce_{0.5}FBiSSe$
6. Low field magnetization and lower critical field of $Sr_{0.5}Ce_{0.5}FBiSSe$

Part 2: Characterization of the $Sr_{0.5}Ce_{0.5}FBiS_{1.5}Se_{0.5}$ compound

1. Sample selected for μSR experiments
2. Magnetic susceptibility of $Sr_{0.5}Ce_{0.5}FBiS_{1.5}Se_{0.5}$

Part 3: Transverse field experiments on the $Sr_{0.5}Ce_{0.5}FBiS_{1.5}Se_{0.5}$ compound

References

Part 1: Characterization of the $Sr_{0.5}Ce_{0.5}FBiSSe$ compound

1. X-ray diffraction

Part of the polycrystalline compound with $x = 1$ was powdered for X-ray diffraction measurements. The powder diffraction pattern is shown in Fig. S1. It was indexed with the P4/nmm space group and lattice parameters a = 4.106 Å and c = 13.476 Å taken from Ref. S1. This confirms the compound crystallizes in the tetragonal $LnOBiS_2$ structure (Ln is lanthanide). The pattern has several smaller peaks (indicated with *) which could not be indexed. These are attributed to the presence of one or more impurity phases. We estimate the amount of impurity phases is of the order of 10 % of the sample volume.

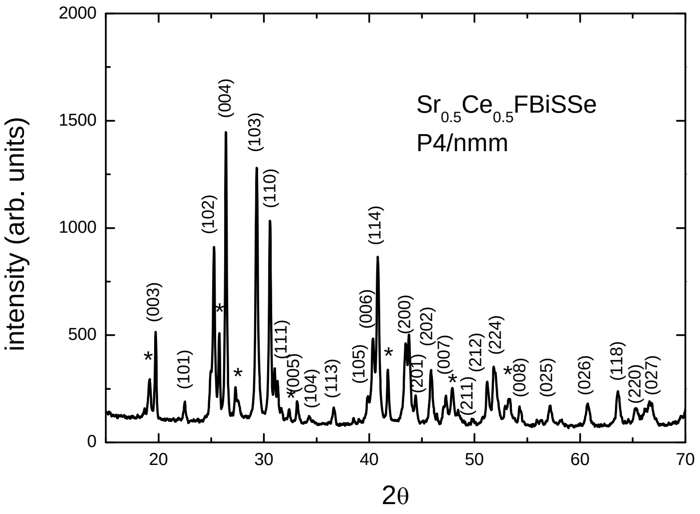

Fig. S1 Powder X-ray diffraction pattern of $Sr_{0.5}Ce_{0.5}FBiSSe$.

2. Magnetic susceptibility and magnetization of $Sr_{0.5}Ce_{0.5}FBiSSe$

The magnetic susceptibility, $\chi(T)$, and the magnetization, $M(H)$, for x = 1.0 were measured in a Physical Properties Measurement System (PPMS) of Quantum Design down to 2.0 K on a bar-shaped sample (1.0×1.5×5 mm^3) with mass 50 mg. The inverse molar susceptibility $1/\chi_M$ measured in a field B = 1 T is a linear function of temperature in the range 70-300 K as shown in Fig. S2. The deduced effective moment, p_{eff}, has a value of 2.35 μ_B/Ce and the paramagnetic Curie Weiss temperature θ_P is -28.1 K. These values are comparable to those reported in the literature: 2.29 μ_B/Ce and θ_P = -8.2 K (Ref. S1). The value of p_{eff} is smaller than the free-ion value 2.54 μ_B for trivalent Ce, which indicates the presence of crystalline electric field effects. The magnetization, measured at fixed temperatures in the range 2-50 K and fields up to 8 T, is shown in Fig. S3. At low temperatures the magnetization saturates in the high field region and attains the large value ~ 0.9 μ_B/Ce at $T = 2$ K. The steep increase in low fields at $T = 2$ K corresponds to a spontaneous magnetization with a ferromagnetically ordered moment m_0 ~ 0.2 μ_B/Ce.

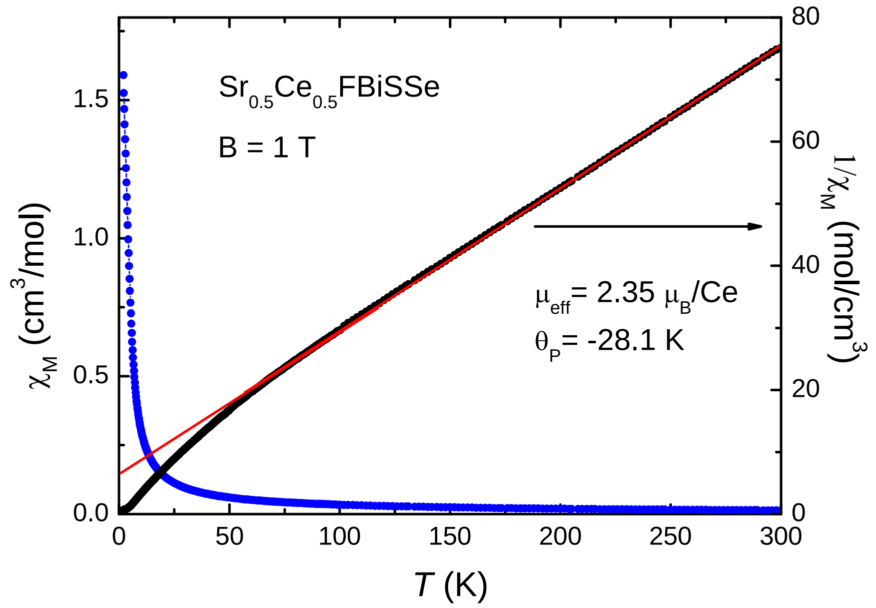

Fig. S2 Left axis: Molar dc-susceptibility of $Sr_{0.5}Ce_{0.5}FBiSSe$ measured in a field of 1 T (blue symbols). Right axis: $1/\chi_M$ (black symbols) and Curie-Weiss fit in the temperature range 70-300 K (red line).

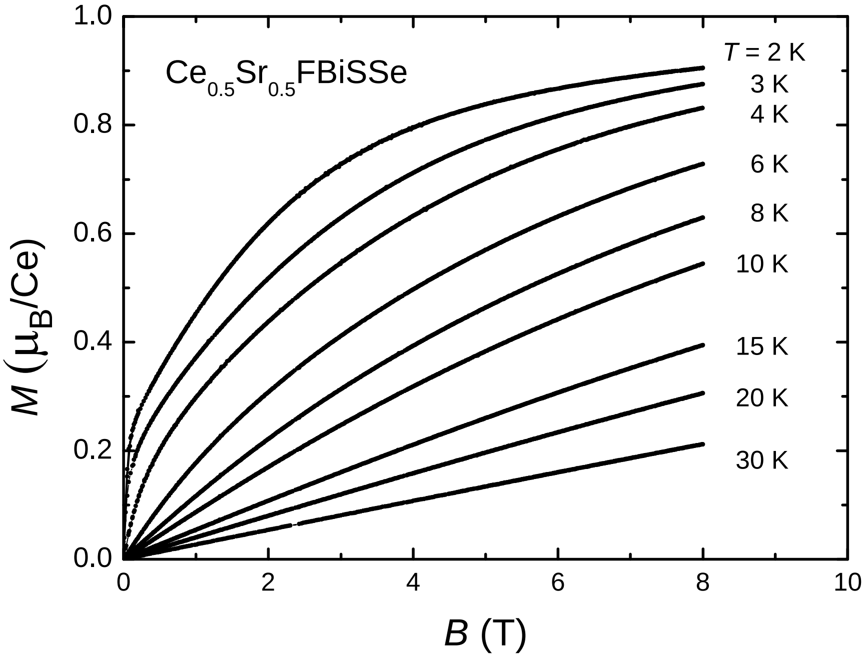

Fig. S3 Magnetization of $Sr_{0.5}Ce_{0.5}FBiSSe$ measured at the indicated temperatures.

3. Specific heat of $Sr_{0.5}Ce_{0.5}FBiSSe$

The specific heat for $x = 1.0$ was measured on a sample with a mass of 30 mg by the relaxation method using the Heat Capacity Option in the PPMS in the temperature range 2-300 K. In Fig. S4 we show the low temperature data ($T < 10$ K) in a plot of c/T versus T^2. The large and broad peak is due to the ferromagnetic order. The Curie temperature is identified by the growing increase of c/T at $T_C = 3.3$ K. The dashed linear line represents $c = \gamma T + \beta T^3$, where γ and β are the usual coefficients for the conduction electron and lattice contributions, respectively. The extracted values $\gamma = 22$ mJ/molK^2 and the Debye temperature $\theta_D = 180$ K are comparable to the values $\gamma = 12$ mJ/molK^2 and $\theta_D = 187$ K reported for $x = 0.5$ in Ref. S1. The magnetic entropy S_m obtained after subtracting the electronic and lattice contributions is shown in the inset. S_m saturates at 0.41×Rln2 at 10 K.

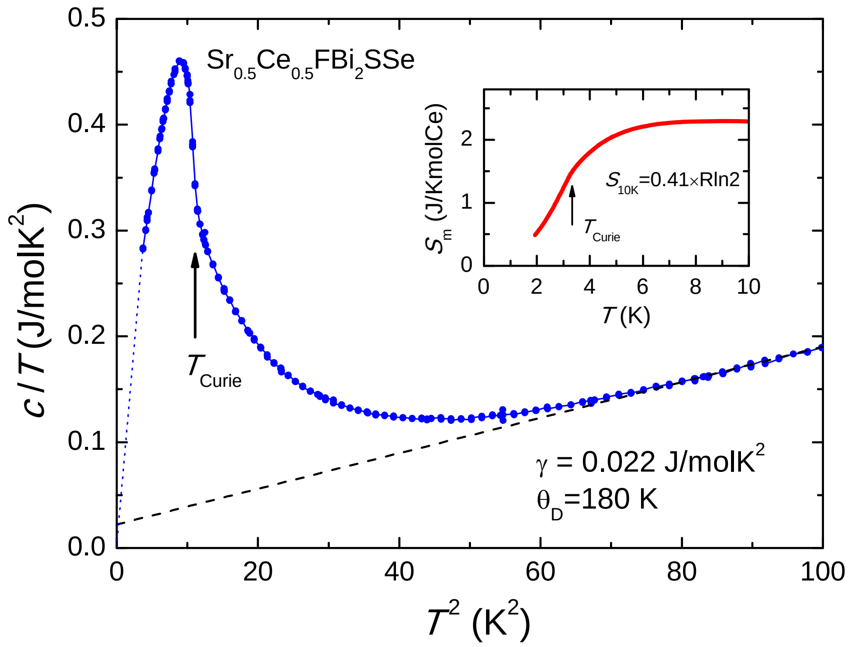

Fig. S4. Specific heat of $Sr_{0.5}Ce_{0.5}FBiSSe$ in a plot of c/T versus T^2. The dashed line represents $c = \gamma T + \beta T^3$. The inset shows the entropy associated with the magnetic transition. The arrows indicate the Curie temperature.

4. Ac susceptibility of $Sr_{0.5}Ce_{0.5}FBiSSe$

The ac-susceptibility, χ_{ac}, was measured on a bar-shaped sample in a driving field of 1 Oe and a frequency f = 1013 Hz in the PPMS in the temperature range 2-10 K. The zero-field cooled in-phase, χ'_{ac}, and out-of-phase, χ''_{ac}, signal are shown in Fig. S5. The increase of χ'_{ac} below ~ 4 K is due to magnetic order. The Curie point as determined by the specific heat data (Fig. S4) is indicated by the arrow. The diamagnetic signal is due to superconductivity, with a transition temperature T_{sc} = 2.92 K as determined by the midpoint of the transition. Close to this temperature χ''_{ac} peaks. The size of the diamagnetic signal measured in 1 Oe corresponds to a superconducting volume fraction of ~ 70 %. Data taken in small applied fields up to 0.075 T show the depression of superconductivity.

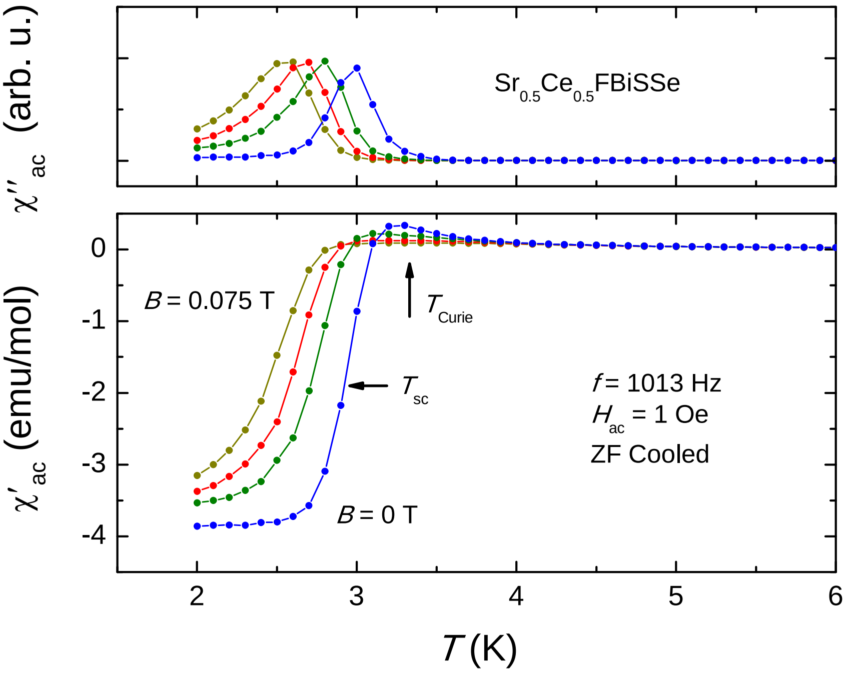

Fig. S5 Ac susceptibility of $Sr_{0.5}Ce_{0.5}FBiSSe$ measured in zero field and small applied fields from right to left B = 0, 0.025, 0.050 and 0.075 T.

5. Electrical resistivity and upper critical field of $Sr_{0.5}Ce_{0.5}FBiSSe$

The electrical resistivity, ρ, was measured in the PPMS in the temperature range 2-300 K. Data were taken on a bar-shaped sample with an excitation current of 1 mA. The results are shown in Fig. S6. The resistivity has a metallic temperature variation. Superconductivity (see lower inset) has an onset temperature of 3.70 K, while zero resistance is observed at 2.89 K, which is close to T_{sc} determined by ac-susceptibility. In the lower inset the superconducting transition in fields between 0 and 1.5 T is reported. The upper critical field, B_{c2}, extracted from resistivity data in fixed fields is shown in the upper inset. Here we follow the same procedure as in Ref. S1 and take data points at 90% of the normal state resistivity value at 4 K ($0.9\times\rho_N$) indicated by the horizontal dashed line in the lower inset. We remark the superconducting transition broadens considerably in field and the $0.9\times\rho_N$ criterion results in an overestimation of B_{c2}. The red line shows a comparison of $B_{c2}(T)$ with the Werthamer, Helfand and Hohenberg (WHH) expression for a weak-coupling spin-singlet orbital-limited superconductor (Ref. S2).

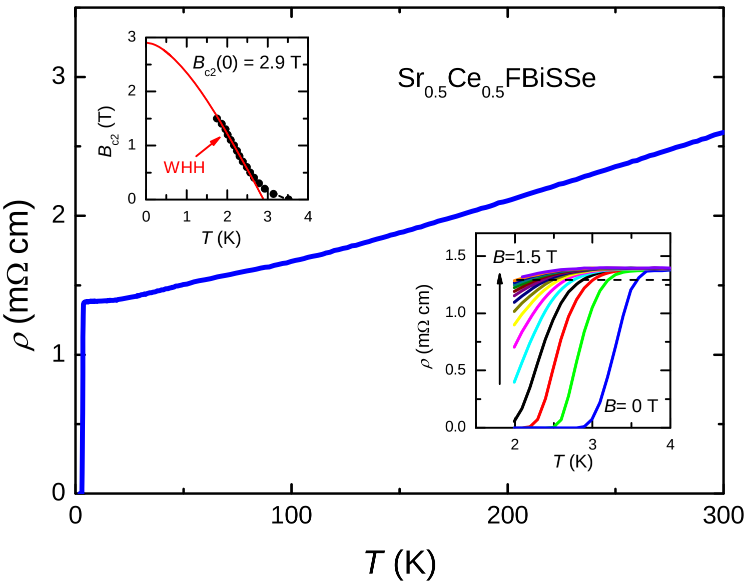

Fig. S6 Electrical resistivity of $Sr_{0.5}Ce_{0.5}FBiSSe$ as a function of temperature. Lower inset: $\rho(T)$ in fixed magnetic fields between 0 and 1.5 T with steps of 0.1 T. The horizontal dashed line represents $0.9\times\rho_N$. Upper inset: Upper critical field $B_{c2}(T)$ (solid symbols). The red line is a comparison with the WHH model.

6. Low field magnetization and lower critical field of $Sr_{0.5}Ce_{0.5}FBiSSe$

In order to investigate the superconducting state the low field magnetization $M(H)$ was measured in the PPMS in the temperature range 1.9 - 4 K, see Fig. S7(a). Upon cooling below 4 K the slope dM/dH first increases due to ferromagnetic order. Below 2.8 K a diamagnetic signal is observed. In the superconducting state, at low fields $M = -H$ and the field where $M(H)$ deviates from a linear function is taken as the lower critical field H_{c1}. $H_{c1}(T)$ follows the usual temperature variation $H_{c1}(T) = H_{c1}(0)\,(1-(T/T_{sc})^2)$ with $H_{c1}(0) = 6$ Oe as shown in Fig. S7(c). In Fig. S7(b) we show a typical magnetic hysteresis loop measured at $T = 2.0$ K. The superconducting loop is superposed on a ferromagnetic loop.

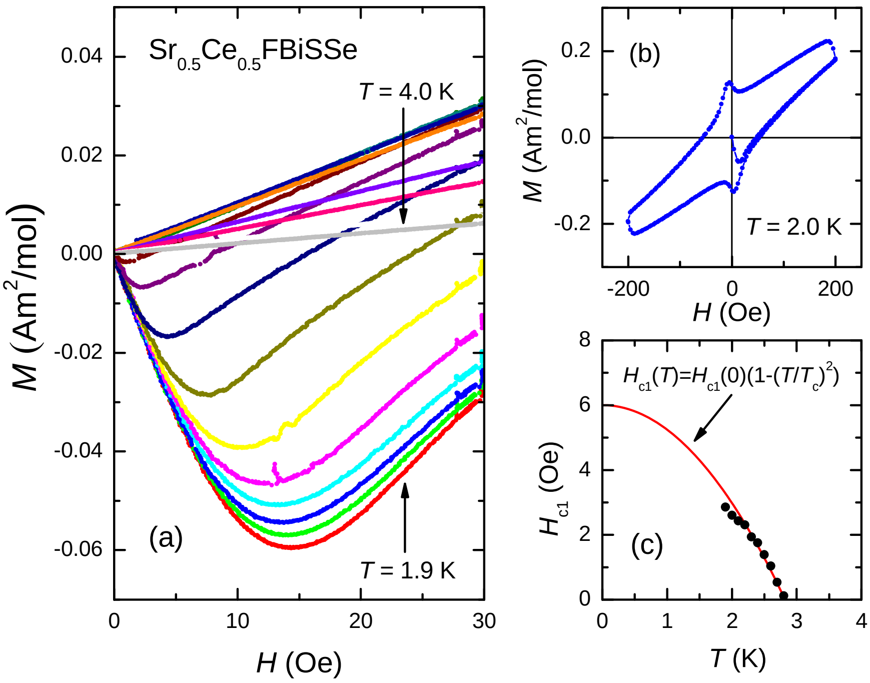

Fig. S7 (a) Low field magnetization of $Sr_{0.5}Ce_{0.5}FBiSSe$ at temperatures of 1.9 - 3.2 K in steps of 0.1 K, and at 3.4, 3.5, 3.75 and 4.0 K. (b) Superposed ferromagnetic and superconducting magnetization loop at 2.0 K. (c) Lower critical field $H_{c1}(T)$ (solid symbols). The red line represents the standard quadratic temperature variation of H_{c1}.

Part 2: Characterization of the $Sr_{0.5}Ce_{0.5}FBiS_{1.5}Se_{0.5}$ compound

1. Sample selected for μSR experiments

The $x = 0.5$ batch was prepared by Thakur *et al.* in India [S1]. Part of the batch was sent to Dresden and cut in several pieces for magnetization, transport and specific heat measurements [S1]. One piece was used for the μSR experiments, but was first characterized by dc-susceptibility measurements (see below). We remark that the different pieces from the same batch give consistent values of the Curie temperature T_C and the superconducting transition temperature T_{sc} and thus are representative for the prepared compound.

2. Magnetic susceptibility of $Sr_{0.5}Ce_{0.5}FBiS_{1.5}Se_{0.5}$

The polycrystalline sample used for the μSR experiments on the $x = 0.5$ compound has been characterized by dc-magnetization measurements in the PPMS in Dresden. Zero field cooled (ZFC) and field cooled (FC) susceptibility data taken in an applied field of 5 Oe are shown in Fig. S8. In the inset a zoom of the data near T_{sc} and T_C is presented. The data are in good agreement with the results reported in Ref. S1.

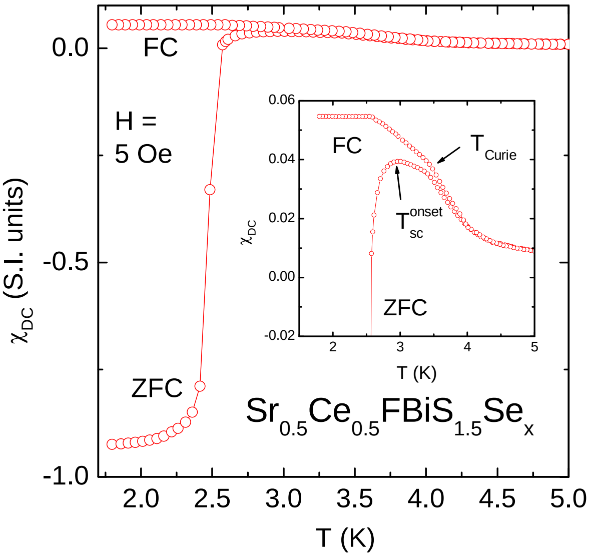

Fig. S8 ZFC and FC dc-susceptibility in S.I. units of $Sr_{0.5}Ce_{0.5}FBiS_{1.5}Se_{0.5}$ measured in a field of 5 Oe. Arrows in the inset point to the Curie temperature $T_C = 3.4$ K and the onset temperature for superconductivity $T_{sc}^{onset} = 2.9$ K.

Part 3: Transverse field experiments on the $Sr_{0.5}Ce_{0.5}FBiS_{1.5}Se_{0.5}$ compound

To estimate the superconducting volume fraction of the sample with x = 0.5 we fit our TF µSR time spectra to a sum of multiple Gaussian components along with a sinusoidally oscillating part above and below the superconducting transition temperature (Fig. S9):

$$A(t) = A_0 \sum_{i=1}^{n} f_i \exp(-\sigma_i^2 t^2 / 2) \cos(\gamma_\mu B_i t + \phi)$$

where A_0 is the initial asymmetry, f_i, σ_i, and B_i are the fraction, the muon relaxation rate, and first moment of the *i*-th component, ϕ is the temperature independent initial phase of the muon-spin ensemble, and γ_μ is the muon gyromagnetic ratio, respectively. (This method is frequently used to analyze a broad field distribution in the superconducting state [S3].) In the paramagnetic state at T = 10 K well above the superconducting and the ferromagnetic transition temperatures the spectrum can be described by a single component with an average field equal to the applied magnetic field B = 100 G (Fig. S9). However, in the superconducting state well below T_{sc} at T = 0.25 K the spectrum is strongly broadened and has an asymmetric shape. In this case we used a four component fit: three components (with average fields different from the applied magnetic field) were necessary to describe the superconducting and ferromagnetic volume fractions and the fourth component with the average field at the applied magnetic field corresponds to the paramagnetic volume fraction. The Fast Fourier Transform in Fig. S9(b) shows that the paramagnetic volume fraction in the superconducting state is about 50 %. This indicates that even in the sample with x = 0.5 the superconducting volume fraction is relatively low but above the percolation threshold p_c ~ 0.2 % [S4]. Therefore, in small applied magnetic fields (below the first critical field) the whole sample volume is screened by the superconducting currents. This explains why the superconducting volume fraction was overestimated using the magnetization or transport data (see Fig. S8 and Thakur *et al*. [S1]).

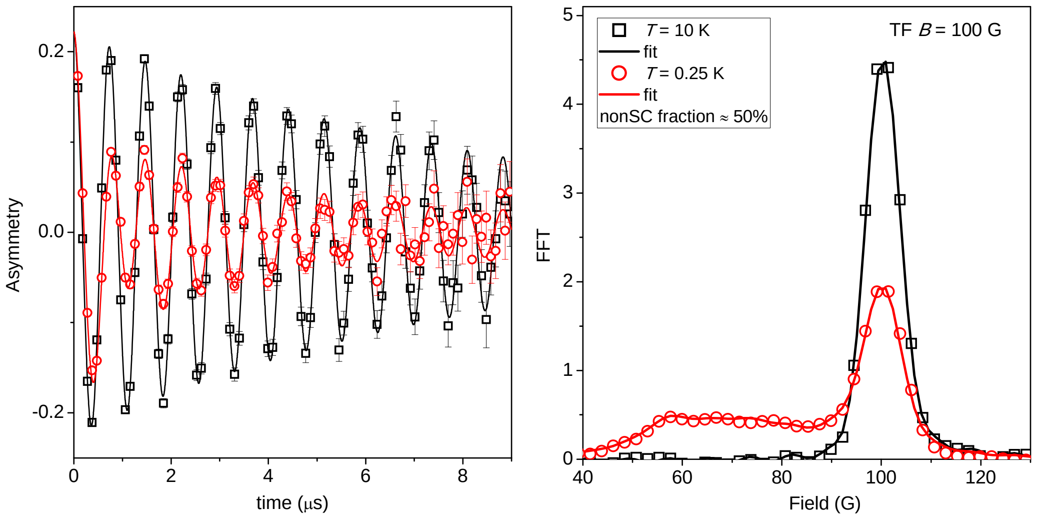

Fig. S9 (a) The transverse-field µSR time spectra of $Sr_{0.5}Ce_{0.5}FBiS_{1-x}Se_x$ (x = 0.5) above and below T_{sc} measured in applied field B = 100 G. (b) Real part of the Fast Fourier Transform (FFT) of the data shown in (a).

References

[S1] G.S. Thakur, G. Fuchs, K. Nenkov, Z. Haque, L.C. Gupta and A.K. Ganguli, Sci. Reports **6**, 37527 (2016).

[S2] N.R. Werthamer, E. Helfand and P.C. Hohenberg, Phys. Rev. **147**, 295 (1966).

[S3] A. Maisuradze, R. Khasanov, A. Shengelaya and H. Keller, J. Phys.: Cond. Matt. **21**, 075701 (2009).

[S4] V. Grinenko, E.P. Krasnoperov, V.A. Stoliarov, A.A. Bush and B.P. Mikhajlov, Solid State Comm. **138**, 461 (2006).
